# Green Production and Interaction of Carboxylated CNTs/Biogenic ZnO Composite for Antibacterial Activity

**DOI:** 10.3390/bioengineering9090437

**Published:** 2022-09-04

**Authors:** Saghir Hussain, Noorulain Khakwani, Yasir Faiz, Sonia Zulfiqar, Zahid Shafiq, Faisal Faiz, Abeer Elhakem, Rokayya Sami, N. I. Aljuraide, Tanveer Farid, Mahmood D. Aljabri, Mohammed M. Rahman

**Affiliations:** 1 Institute of Chemical Sciences, Bahauddin Zakariya University, Multan 60800, Pakistan; 2 Department of Chemistry, Khwaja Fareed University of Engineering & Information Technology, Abu Dhabi Road, Rahim Yar Khan 64200, Pakistan; 3 Chemistry Division, Directorate of Science, Pakistan Institute of Nuclear Science & Technology (PINSTECH), Islamabad 45650, Pakistan; 4 Department of Chemistry, Faculty of Science, University of Ostrava, 30. Dubna 22, 701 03 Ostrava, Czech Republic; 5 State Key Laboratory of Analytical Chemistry for Life Science, School of Chemistry & Chemical Engineering and Center of Materials Analysis, Nanjing University, Nanjing 210023, China; 6 Department of Biology, College of Science and Humanities in Al-Kharj, Prince Sattam Bin Abdulaziz University, Al-Kharj 11942, Saudi Arabia; 7 Department of Food Science and Nutrition, College of Sciences, Taif University, P.O. Box 11099, Taif 21944, Saudi Arabia; 8 Department of Physics, Turabah University College, Turabah Branch, Taif University, P.O. Box 29717, Taif 21944, Saudi Arabia; 9 School of Chemical Engineering, Nanjing University of Science and Technology, Nanjing 210094, China; 10 Department of Chemistry, University College in Al-Jamoum, Umm Al-Qura University, Makkah 21955, Saudi Arabia; 11 Department of Chemistry, Faculty of Science, King Abdulaziz University, Jeddah 21589, Saudi Arabia

**Keywords:** green chemistry, ZnO nanoparticles, carbon nanotubes, nanocomposite, antibacterial activity, biogenic synthesis

## Abstract

Using biomolecule-rich plant extracts, the conversion of metal ions to metal oxide nanoparticles via abiogenic approach is highly intriguing, environmentally friendly, and quick. The inherent inclination of plant extracts function as capping agents in the insitu synthesis. In this study, biogenic zinc oxide nanoparticles (ZnO−NPs) were synthesized using an aqueous leaf extract from *Moringaoleifera*. The ZnO−NPs were then mixed with carboxylated carbon nanotubes (CNTs) to create a carboxylated CNTs/biogenic ZnO composite using asol–gel method. The CNTs/ZnO composite displayed 18 mm, 16 mm, and 17 mm zones of inhibition (ZOI) against *Bacillus cereus*, *Pseudomonas aeruginosa*, and *Escherichia coli*, respectively. In contrast with ZnO−NPs, the produced carboxylated CNTs/ZnO composite demonstrated a 13 percent elevation in ZOI as antibacterial activity against *Bacillus cereus* ATCC 19659, *Escherichia coli* ATCC 25922, and *Pseudomonas aeruginosa* ATCC 27853. The characterization of ZnO−NPs and the carboxylated CNTs/ZnO composite were performed via FTIR, UV/Vis spectroscopy, SEM, and XRD. The XRD pattern depicted a nano−sized crystalline structure (Wurtzite) of ZnO−NPs and a carboxylated CNTs/ZnO composite. The current work comprehends a valuable green technique for killing pathogenic bacteria, and gives fresh insights into the manufacture of metal oxide composites for future research.

## 1. Introduction

The green synthesis of metal oxide nanoparticles (NPs) is considered to be a more efficient and feasible technique than chemical synthesis, as it is a simple method with a good yield and comprises a biological route for NPs production. Green chemistry is also termed as being sustainable chemistry interrelated with the elimination of environmental hazards such as microbes and pathogens, pollutants, and toxic chemicals. The chemical route of synthesis requires the extensive practice of high temperature, pressure, and toxic substances, which are against the norms of a healthier environment [1,2,3]. The *Moringa oleifera* plant is eaten as a vegetable because of its phytoconstituents, such as protein, amino acid, folic acid, nicotinic acid, riboflavin; vitamins A, B, C and E; a number of phenolic compounds, omegas 3 and 6, and many more. These phytoconstituents act as bioactive organic compounds such as alkaloids, flavonoids, glycosides, phenols, saponins, tannins, terpenoids, reducing sugars, and triglycerides [4]. Most of the previous studies on *Moringa oleifera* have shown that the presence of phenolic compounds contributes to different antimicrobial and antioxidant properties [5,6,7,8,9]. Furthermore, the extraction of these phytochemicals through different solvents shows a very effective inhibition activity against microbes [10].

**Figure 1:**
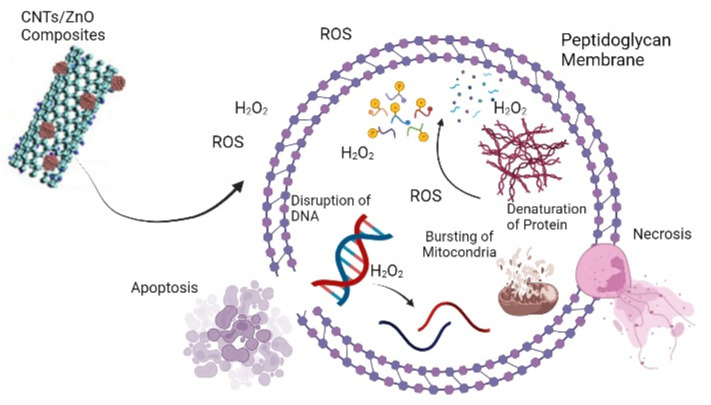
Proposed mechanism of CNTs/ZnO composite antibacterial activity.

The antibacterial property of metal oxide NPs has been advantageous because of their nano−dimensions and extensive surface area−to−volume ratio (SA:V), which enhances interactions directly with microbial membranes [11]. Zinc oxide (ZnO) naturally possesses a wide range of antibacterial and antifungal properties, and the Food and Drug Administration (FDA) of the United States has classified ZnO as being a generally recognized safe substance (21CFR182.8991) [12,13]. Because of their strong antibacterial capabilities, nanocomposite materials comprising ZnO−NPs have been shown to be beneficial in the medical, cosmetic, and food industries. ZnO−NPs have surfaced as efficient antibacterial agents in recent years due to their good performance against strains that are resistant to antibiotics, poor bioavailability, and heat tolerance [14]. ZnO−NPs have been found to have superior antibacterial activities to their micro−sized counterparts, due to a higher degree of penetration and breakdown of the bacterial membrane upon contact. Previously reported studies have hypothesized that ZnO−NPs antibacterial action is caused by hydrogen peroxide (H_2_O_2_), which is produced after the interaction of ZnO NPs with the bacterial cell membrane. Further, H_2_O_2_ acts on the bacterial structure, and causes the oxidative breakdown of the cell structure [15,16] (Figure 1). As a result, the most significant factor in antibacterial action is the formation of H_2_O_2_, but it has also been proposed that electrostatic forces may be responsible for ZnO−NPs adhesion to microbial surfaces [2].

Due to their exceptional physicochemical, electrical, and mechanical characteristics as a nanomaterial, and their potential to develop new applications, carbon nanotubes (CNTs) are crucial [17]. It is widely known that the dimensions of CNTs, including their size and surface area, play a significant role in bioactivity. Specifically, the smaller sized CNTs exhibit a greater surface area, promoting and favoring their biological contact with microbial cells [18,19]. In addition, the biocompatibility of CNTs is one of the characteristics that contributes to the material’s reduced cellular cytotoxicity. In biological toxicity-related studies, functionalized CNTs with different biomolecules such as poly-lysine have shown very low or insignificant toxicological environmental impacts [20,21]. Based on previous studies, the CNT composites with metal and metal oxide nanoparticles exhibited excellent antimicrobial activities. The antibacterial activity of CNTs covered with a silver layer was found to be as good as that of a silver-coated pyrolytic carbon sample [22,23,24]. Previously, composites of ZnO/graphene oxide have been used as surface coatings in medical devices to limit bacterial growth [25]. Therefore, the fabrication of CNTs with various metal oxide-NPs makes them more feasible for use in the treatment of cancer and other biotic applications [26,27].

The purpose of this work was to investigate the green synthesis of ZnO−NPs, as well as the preparation of a carboxylated CNTs/biogenic ZnO composite and its application in combating a variety of bacteria. Briefly, ZnO−NPs were synthesized by stirring Zinc acetate salt with aqueous *Moringa oleifera* leaf extract, followed by extensive washing and stirring at high temperature. The CNTs/ZnO composite was prepared by thoroughly mixing the acid-treated CNTs with ZnO−NPs. The synthesized ZnO−NPs and the CNTs/ZnO composite were characterized using energy-dispersive x-ray (EDX) spectroscopy, Fourier transform infrared (FTIR) spectroscopy, scanning electron microscopy (SEM), UV-Vis spectroscopy, and X-ray diffraction (XRD) analysis. The antibacterial action of the ZnO−NPs and the carboxylated CNTs/ZnO composite were performed through the agar well method, and zones of inhibition (ZOI) were measured. When compared to the antibacterial activity of ZnO−NPs, the CNTs/ZnO composite demonstrated significantly more effective performance.

## 2. Materials and Methods

### 2.1. Research Methods

CNTs were purchased from Sigma Aldrich, and >95% pure zinc acetate dihydrate (99.9%) was purchased from Merck. All the chemicals were of analytical grade and were used without further modification. The samples were prepared by using a ceramic hot plate (Corning PC-420 Dlab ms7-H550-pro), mechanical shaker (IRM ECO 0S10, RPM = 100 per hour), and ultrasonication (Elma Sonic Tasy 30H); and were investigated by using a ZEISS LEO SUPRA 55 field emission scanning electron microscope (FESEM) and energy dispersive X-ray spectroscopy (EDS). The X-ray diffraction (XRD) patterns were examined using a Bruker D8 Advance X-ray powder diffractometer (Karlsruhe, Germany) with Cu/Kα-strokes operation at 40 kV and 40 mA with the 2θ angles of 5–80° at room temperature. Fourier transform infrared spectroscopy (Cary 600 series FTIR Spectrometer, Agilent TechnologiesInc., Santa Clara, CA, USA) was also performed. The sample for FTIR was prepared by taking a small amount of nanocomposites sample (20%) and mixing it with potassium bromide (80%) to make a semitransparent disc, which was then analyzed using a FTIR spectrometer in the wave number range from 500–4000 cm^−1^.

### 2.2. Collection of Moringa oleifera Extract

*Moringa oleifera* leaves were collected locally, and after washing, 20 g of these freshly cut leaves were placed in 100 mL of deionized water for the preparation of the plant extract. The mixture was stirred at 50 °C for 30 min. Then, the filtered mixture was removed and the extract was cooled at room temperature. A pure aqueous extract of phytochemicals was prepared. This aqueous extract was stored at 4°C in a refrigerator and used for metal oxide nanoparticle synthesis.

### 2.3. Synthesis of ZnO−NPs

For the preparation of 0.1 M of zinc acetate salt, 100 cm^3^ of water was mixed with 20 cm^3^ of properly dissolved 0.1 M zinc salt solution. A total of 60 cm^3^ aqueous plant extract solution was added, and the mixture was mixed on a magnetic stirrer and refluxed at 120 °C for 2 h. The color of the mixture changed from green to light brown. Upon completion of the reaction, the mixture was covered with aluminum foil and kept at room temperature for 18 h. The ZnO−NPs settled down at the bottom of the flask in a sol–gel system. After decantation of the filtrate, the remaining portion containing the ZnO−NPs was separated through micro-filter paper. The product was washed thoroughly with distilled water and ethanol, and dried. The prepared nanomaterial was calcinated at 500 °C for 4 h, followed by grinding before further use.

### 2.4. Preparation of the CNTs−COOH/ZnO Composite

Firstly, CNTs were functionalized with the carboxylic group. In this process, CNTs (200 mg) were added in a specific ratio of HNO_3_ and H_2_SO_4_ (60 cm^3^:10 cm^3^) to a medium round bottom flask and refluxed for 48 h at 120 °C [28]. After the stipulated time, the mixture was cooled to ambient temperature before filtration, and washed with deionized water. The blackish colored precipitates were isolated as CNTs−COOH. Secondly, 40 mg of functionalized CNTs was added to 100 cm^3^ of ethanol in a round bottom flask. The mixture was sonicated for 2 h for proper dispersion of the CNTs. After this, 80 mg of ZnO−NPs were added to the CNTs mixture, and this mixture was placed onto a mechanical shaker for 2 h to make a sol–gel system. Next, the entire mixture was placed into a sonication bath for proper dispersion of the composite. The mixture was dried, and 100 mg of composite was achieved from the calcination process at 120 °C.

### 2.5. Antibacterial Action

The antibacterial activity of ZnO−NPs and the CNTs/ZnO composite was evaluated through the agar well method. For this purpose, an inoculum of bacterial strains, i.e., *Bacillus cereus* ATCC 19659, *Escherichia coli* ATCC 25922,and *Pseudomonas aeruginosa* ATCC 27853, was prepared in sterilized nutrient broth. Nutrient agar was prepared, and after sterilization, poured into Petri plates. After solidification, wells (6 mm) were made using a sterilized steel borer. The inoculum of each bacterial strain was spread onto separate solidified nutrient agar plates using a sterilized cotton swab. Then, 200 μL of test samples, i.e., ZnO−NPs and CNTs/ZnO, were added to the respective wells. A 200 μL volume of normal saline solution was used as a negative control. Subsequently, the plates were incubated at 30 °C overnight, and after incubation, the inhibition region was evaluated (in millimeters diameter). The experiment was repeated 3 times to obtain more accurate data.

### 2.6. Statistical Analysis

The data in this report are expressed in the form of mean ± standard deviation (SD) for *n* = 3. The calculations were performed through a standard statistical package for social sciences (SPSS).

## 3. Results and Discussion

### 3.1. Characterization of CarboxylatedCNTs/ZnOComposite

**Figure 2:**
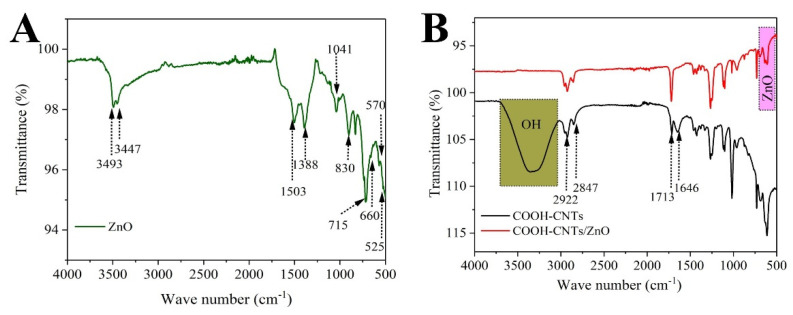
FTIR spectra of (**A**) biogenic ZnO−NPs and (**B**) carboxylated CNTs and CNTs/ZnO composite.

The characterization of the ZnO−NPs and CNTs−COOH/ZnO composite was achieved using different spectroscopic techniques, i.e., EDX, FTIR, SEM, and XRD. The FTIR spectra of CNTs−COOH, ZnO, and CNTs/ZnO are shown in Figure 2. The ZnO peaks are obvious between 500 to 700 cm^−1^. The shoulder at 525 cm^−1^ is a local vibration mode related to donor defects or oxygen vacancies and zinc interstitials. The absorption at 570 cm^−1^ corresponds to oxygen deficiencies in the ZnO structure. The stretching peak at 660 cm^−1^ indicates the vibrations of ZnO−NPs [29,30,31,32]. The peaks appearing at 715 and 830 cm^−1^ represent C-H stretching from alkanes, C=C-H stretching from alkenes, -OH stretching of intramolecular H-bonds, and C=O and C-H stretching from alkanes. The peak at 1388 cm^−1^ represents the unreacted ketone groups showing the presence of flavonoids in ZnO NPs. The peak at 1041 is related to the C-N stretch of an amine functional group. The peaks at 4393 and 3447 cm^−1^ represent O-H stretching vibration in alcohols/phenols in leaf extracts. The extra peaks, i.e., 715, 1388, and 830 cm^−1^ are attributed to the organic molecules acting as a capping agent on ZnO−NPs, and some leaf extract residue left in ZnO−NPs [32,33]. In the FTIR spectrum of the CNTs−COOH, the observed peaks at 1713 cm^−1^ areattributed to the C=O group. The broadband at 3000-3600 cm^−1^ confirms the presence of hydroxyl group in the CNTs−COOH. The appearance of the peak at 1646 cm^−1^ indicatesa C=C double bond backbone for CNTs. The peaks at 2847 and 2922 cm^−1^ are attributed to the stretching vibrations of the CH_2_/CH_3_ groups present at the carboxylated CNTs [34]. The FTIR spectrum of the CNTs/ZnO composite shows the presence of ZnO−NPs via strong absorption between 500 to 700 cm^−1^, along with the absorbance peaks from the leaf residue present in NPs. Furthermore, the characteristic peaks of CNTs−COOH can also be traced in composite to1713, 2847, and 2922 cm^−1^. So, this spectrum confirms the presence of both CNTs and ZnO−NPs incomposite [35].

**Figure 3:**
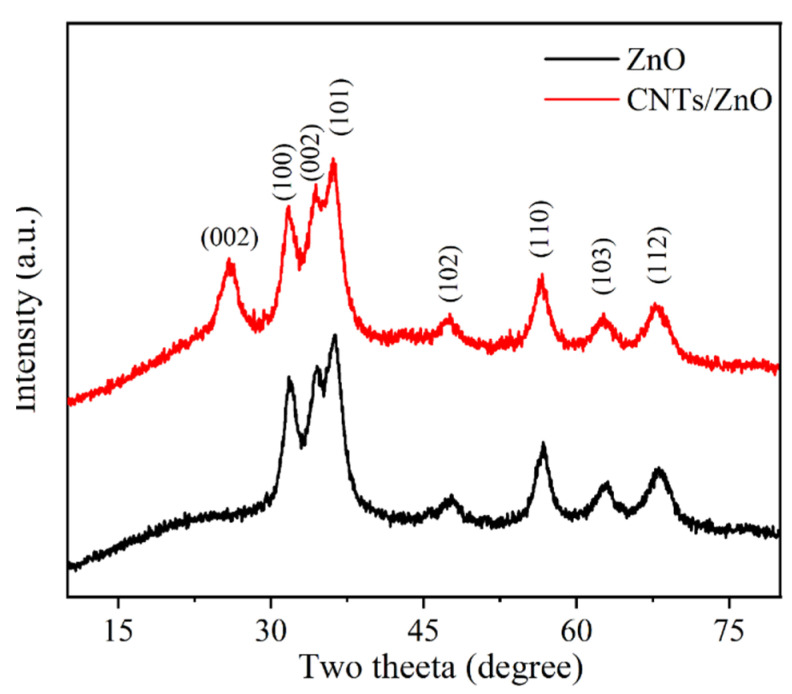
XRD analysis of ZnO−NPs and CNTs/ZnO composite.

**Figure 4:**
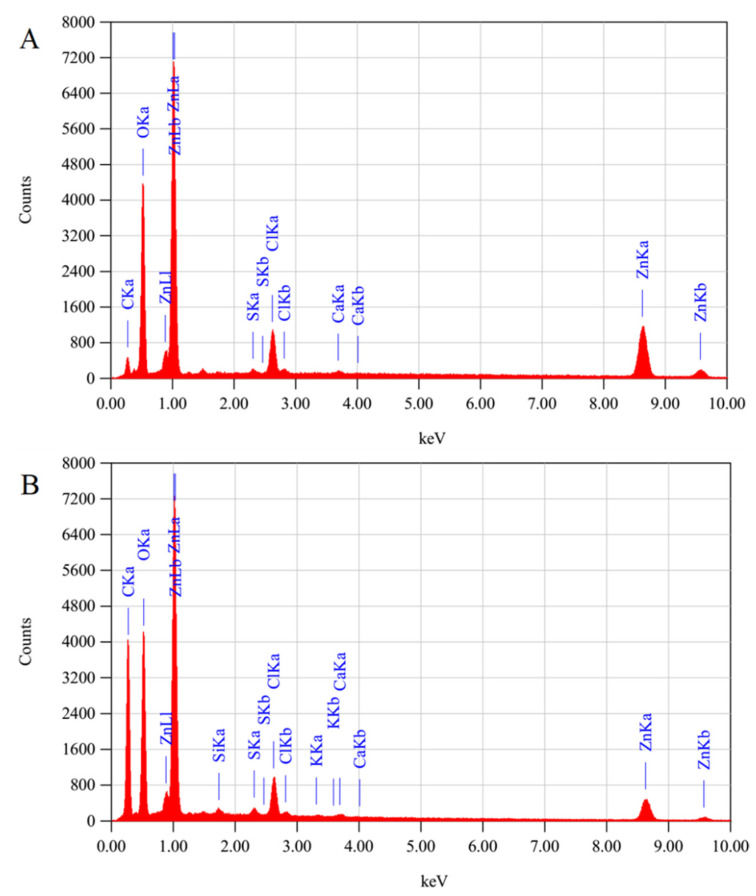
EDX spectra of (**A**) ZnO−NPs, along with the EDX spectra of (**B**) CNTs/ZnO composite.

The structural properties of prepared ZnO−NPs were demonstrated using an X-ray diffractometer (XRD) with Cu Kα radiations (λ = 1.5406 Å). In Figure 3, the obtained XRD patterns of CNTs/ZnO and ZnO−NPs revealed that the diffraction peaks at diffraction angles (2θ°) of 31.73°, 34.46°, 36.06°,47.6°, 56.6°, 62.6°, and 67.7° matched well with the hexagonal wurtzite ZnO polycrystalline structure and agreed with the reflection from the crystal planes (100), (002), and (101), (102), (110), (103), and (112), respectively. The XRD data is in accordance withthe JCPDS reference code 00-036-1451 [36,37].The reflection from the crystal planes of carboxylated CNTs is present at diffraction angles (2θ°) of 25.88° assigned to the (002) plane, due to which the intensity of the peak is increased comparatively, and the existence of carboxylated CNTs is confirmed. The Scherer formula was used to estimate the crystallite size of the ZnO−NPs for the FWHM of the well-oriented peak of diffraction, which was obtained at about 35 nm. The XRD pattern of ZnO and the CNTs/ZnO composite shows some peak shifts from the standard JCPDS 36-1451 due to the high amount of metal ions, especially calcium, being present in *Moringa oleifera*. These metal ions are expected to contribute to the doping of the ZnO crystalline structure [38,39]. In Figure 4, the EDX spectrum clearly depicted the elements of O and Zn in the sample, and thus evidenced the presence of ZnO−NPs. The presence of ZnO−NPs in carboxylated CNTs was confirmed by the EDX spectrum, which showed that the elements C, Zn, and O exist iin the produced composite particles.

**Figure 5:**
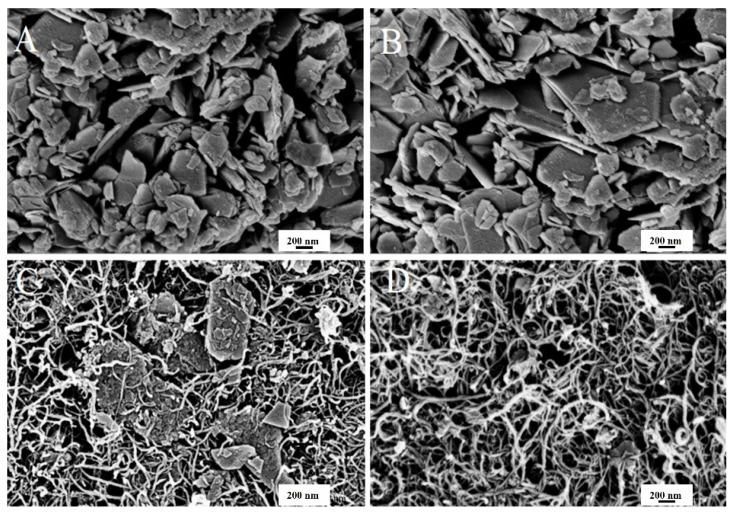
SEM images. (**A**,**B**) ZnO−NPs and (**C**,**D**) CNTs/ZnO composite.

SEM was used to observe the morphology of the prepared ZnO−NPs and CNTs/ZnO composite. Figure 5A,B shows the SEM scan of the flakes of ZnO−NPs and CNTs/ZnO formed through green synthesis. It can be clearly observed that ZnO has a plate-like structure, with a size ranging from nanometers to micrometers. The addition of ZnO into CNTs provided a mixed morphology of composites, where the ZnO particles embed viaa highly conductive CNTs network. This network enables the ZnO−NPs particles to perform effective antibacterial activities.

### 3.2. Antibacterial Activity of ZnO and CNTs/ZnOComposite

**Figure 6:**
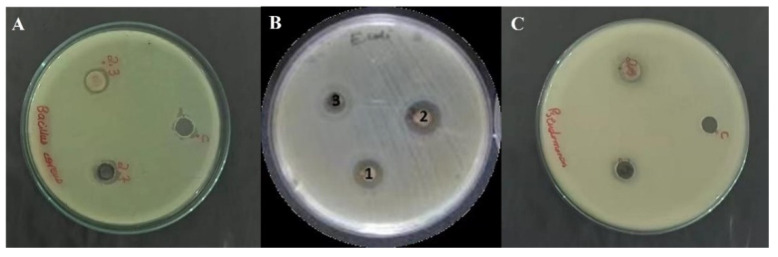
Antibacterial activity of ZnO-NPs and CNTs/ZnOcomposite on (**A**) *Bacillus cereus*, (**B**) *Escherichia coli*, and (**C**) *Pseudomonas aeruginosa*.

Green synthesized ZnO−NPs and their composites with CNTs were subjected to antibacterial activity through the agar well method [40]. The results of this activity showed that the CNTs/ZnO composite has greater antibacterial potential against *Bacillus cereus* and *Pseudomonas aeruginosa*, while the potential against *Escherichia coli* was moderate, as shown in Figure 6. However, in comparison to the ZnO−NPs, the CNTs/ZnO composite showed slightly higher zones of inhibition (Figure 6).

Previously reported studies have also investigated the antibacterial properties of natural plant sources [41,42,43]. Furthermore, the antibacterial activities of ZnO−NPs and their composites have also been extensively studied. In another study, a change in the proportion of Ag metal in Ag-doped ZnO−NPs alters the MIC against *Staphylococcus aureus*. It was discovered that bacterial susceptibility is directly related to the silver content, and inversely related to the crystalline dimension [44]. Another study found that ZnO−NPs generated by *Olea europaea* had an inhibitory zone of 2.2 cm against *Xanthomonas oryzae pv*. The same study found that ZnO−NPs produced through *Lycopersicon esculentum* and *Matricaria chamomilla* showed a different inhibitory potential against Oryzae (Xoo) strain GZ 0003 [32].

Another work by Bhuyan et al. found the antibiotic property of green synthesized ZnO increases with the increase in concentration of NPs [45]. However, in another study, it has been found that ZnO−NPs synthesized from the extracts of four plants, *Betavulgaris*, *Brassica oleracea* var. *Italica*., and *Cinnamomum tamala* and *verum,* and ZnO−NPs prepared from *Beta vulgaris*, were ineffective against *Staphylococcus aureus* [46]. This study supports our findings that although our ZnO−NPs and CNTs/ZnO composite did not show significant antibacterial activity, they did show moderate antibacterial potential. ZnO−NPs and CNTs/ZnO composites can destroy bacterial strains, as NPs can easily penetrate the cell membrane and disrupt the DNA structure by releasing hydrogen peroxide that undergoes an oxidative breakdown of the bacterial cell structure [47,48,49].

Table 1 explains the ratio of the inhibition zone of the ZnO−NPs and CNTs/ZnO composite.

The zones of inhibition of the ZnO−NPs and the carboxylated CNTs/ZnO composite resulted in a range of 14–16 mm and 16–18 mm, respectively, on nutrient agar plates against *Escherichia coli*, *Bacillus cereus*, and *Pseudomonas aeruginosa* (Table 1).

Individually, CNTs and ZnO−NPs have good antimicrobial potentials, but both have some limitations in their actions. For example, the CNTs have greater cytotoxicity for biological cells, and their mode of action is also sometimes vigorous enough to destroy useful content as well. However, the penetration of and surface attachment of the ZnO−NPs is not as effective as those of the CNTs. Thus, the hybrid of both species, the CNTs/ZnO composite, can reduce the limitations of both substances and enhance their good characteristics. Further studies should be carried out to check the minimum inhibitory concentration (MIC) and antibacterial action of the CNTs/ZnO composite against more bacterial species.

## 4. Conclusions

The metal oxide composite of CNTs showed more antibacterial potential as compared to metal oxide NPs. The focus of the present research work was on the green synthesis of the ZnO−NPs and then a comparison of its biological activities with a CNTs composite. The green synthesis of ZnO−NPs and its composite were confirmed using cutting-edge spectrometric characterization techniques, i.e., EDX, FTIR, SEM, and XRD. The antibiotic activity of the CNTs/ZnO composite combination was found to be more efficient against *Bacillus cereus*, *Escherichia coli*, and *Pseudomonas aeruginosa*, as compared to the ZnO-NPs.

## Figures and Tables

**Table 1:** ZOI range of ZnO−NPs and CNTs/ZnO on bacterial species.

Bacterial Specie	ZOI of ZnO−NPs	ZOI of CNTs/ZnO Composite
	(mm)	(mm)
*Escherichia Coli*	18.4 ± 3.7	18.8 ± 3.7
*Bacillus Cereus*	13.9 ± 0.9	16.8 ± 2.1
*Pseudomonas aeruginosa*	16.3 ± 1.5	17.2 ± 1.4

## Data Availability

Not applicable.
